# Relationship between bleeding risk and arterial stiffness in patients with cerebral aneurysms

**DOI:** 10.55730/1300-0144.5959

**Published:** 2025-01-11

**Authors:** Mehmet Selim GEL, Hasan Çağrı POSTUK, İskender Samet DALTABAN, Savaş ÖZER, Ercan AYDIN, Emrah KESKİN

**Affiliations:** 1 Department of Neurosurgery Clinic, Trabzon Kanuni Training and Research Hospital, Trabzon, Turkiye; 2 Department of Neurosurgery Clinic, Ankara Alife Hospital, Ankara, Turkiye; 3 Department of Cardiology Clinic Trabzon Kanuni Training and Research Hospital, Trabzon, Turkiye; 4 Department of Neurosurgery Clinic, Zonguldak Bülent Ecevit University Hospital, Zonguldak, Turkiye

**Keywords:** Arterial stiffness, cardio-ankle vascular index, cerebral aneurysm, subarachnoid haemorrhage

## Abstract

**Background/aim:**

A comprehensive risk factor assessment evaluating the susceptibility of cerebral aneurysms (CAs) to rupture has not yet been established. Therefore, the clinical management of unruptured CAs remains uncertain. This study aimed to assess whether arterial stiffness was associated with rupture risk in patients with CAs.

**Materials and methods:**

Following magnetic resonance angiography, 49 patients with CAs and subarachnoid haemorrhage and ruptured CAs (confirmed via digital subtraction angiography) were included in the study. Arterial stiffness was measured using the VaSera VS-1000 vascular scanning system and expressed as cardio-ankle vascular index (CAVI) values. The CAVI values were compared between the patient groups.

**Results:**

The mean age of the cerebrovascular aneurysm group was 51 ± 11 years, while that of the cerebrovascular aneurysmal haemorrhage group was 58 ± 12 (p = 0.308) years. Left and right CAVI values were significantly higher in the cerebrovascular aneurysm group (p < 0.05 for both). The CAVI values were positively correlated with haemorrhagic CA (p < 0.05).

**Conclusion:**

This study revealed that increased arterial stiffness was associated with an increased risk of haemorrhage in patients with CAs. This result demonstrates the importance of evaluating arterial stiffness as an informative parameter for treatment and follow-up decisions in patients with CAs.

## 1. Introduction

The most common cause of spontaneous subarachnoid haemorrhage (SAH) is intracranial aneurysms (IAs). These aneurysms are defined as irreversible, pathological focal enlargements of the vessel wall, with a prevalence of 1%–2% in the population [1]. With the widespread use of cross-sectional imaging methods, the detection rate of unruptured aneurysms has increased, altering the approach to managing these patients. The annual risk of rupture in IAs is 1.4%, and the five-year risk of rupture is 3.4%. Independent risk factors for rupture include the patient’s age, aneurysm size, arterial hypertension, aneurysm localisation, and a previous history of SAH [2].

The mortality rate of aneurysmal SAH remains between 30% and 40%, despite advancements in intensive care, and morbidity among survivors is approximately 30% [3]. Therefore, treating IAs before rupture is crucial. In patients with aneurysms smaller than 7 mm and no risk factors, the rupture risk is low, and these patients are typically followed-up conservatively. However, there is no consensus on the treatment of unruptured IAs larger than 7 mm [4].

Cumulative arterial wall disruption due to degeneration and remodelling, accompanied by inflammatory cell infiltration, has been associated with the development of cerebral aneurysm (CA) rupture in several studies [5,6]. Altered arterial wall elastic properties and hypertrophic remodelling may predispose CAs to rupture [7,8]. The cardio-ankle vascular index (CAVI), a parameter used to assess arterial stiffness, is a reliable and practical index of blood pressure-independent arterial stiffness and encompasses both organic and functional stiffness [9,10]. Compared to other methods for assessing arterial stiffness, CAVI measurement is notable for being easily applicable, reproducible, operator-independent, and less affected by blood pressure variations [11].

This study aimed to investigate the hypothesis that arterial stiffness, as measured by the CAVI, may predict the risk of CA rupture in patients with CAs. The study design addressed a novel aspect that has not been previously reported in the literature.

## 2. Materials and methods

### 2.1. Study population

Two separate groups diagnosed with CAs and ruptured CAs between September 2023 and September 2024 were evaluated consecutively. The CA group was selected based on inclusion and exclusion criteria from patients presenting to the neurosurgery outpatient clinic with headaches, whose diagnoses were confirmed via digital subtraction angiography (DSA) following detection of a CA on magnetic resonance angiography. Patients with ruptured CAs were consecutively selected according to the inclusion and exclusion criteria from those referred from the emergency department with a diagnosis of subarachnoid haemorrhage, confirmed by DSA imaging. Patients with aneurysms in the anterior circulation were included, while those with aneurysms in the posterior circulation were excluded. Only patients with aneurysm sizes of 10 mm or less were included.

The exclusion criteria were a history of atherosclerotic heart disease and/or myocardial infarction, neurological deficits, diabetes mellitus, heart failure (left ventricular ejection fraction <50%), renal failure (estimated glomerular filtration rate <60 mL/min/1.73 m^2^), peripheral arterial disease, ankle-brachial index (ABI) <0.9, and severe comorbid conditions. After applying these criteria, 49 patients were prospectively included in the study. Of the patients included, 29 were treated microsurgically, and 20 underwent endovascular treatment. The patients with CAs were divided into two groups: the cerebrovascular aneurysm group and the cerebrovascular aneurysmal haemorrhage group. Baseline demographic data, biochemical parameters, and CAVI values were compared between the groups.

The study was conducted in accordance with the principles outlined in the Declaration of Helsinki, and ethical consent was obtained from the local ethics committee (number: 2024-99).

#### 2.1.1. Assessment of arterial stiffness

Arterial stiffness was assessed using the CAVI. Measurements were performed using the VaSera VS-1000 system (Fukuda-Denshi Co., Ltd., Tokyo, Japan) [12]. The CAVI is determined by recording the distance from the aortic valve to the measurement point at the ankle and the time delay between the closure of the aortic valve and the detected pressure wave change.

The patients were placed in the supine position with cuffs and clamps attached to their upper arms and ankles. After a rest period, the CAVI was calculated automatically using electrography, phonocardiography, and measurement of the pressure and waveforms of the brachial and ankle arteries.

There are differences in blood pressure between the heart and the ankle. The CAVI blood pressure was calculated with the left CAVI (L-CAVI) value using the right upper arm and left lower leg blood pressure, and right CAVI (R-CAVI) using the left upper arm and right lower leg blood pressure values when only the left upper arm was available due to physical difficulties on the right side [13]. The ABI measurement is found by dividing the systolic pressures of the lower extremities by the value with high pressure in the upper extremities. The ABI ≤0.90 at rest is significant for the PAH, it is 99% exclusionary for >1.10 PAH of the ABI measurement [14].

### 2.2. Statistical analysis

The normality of the data distribution was evaluated using the Kolmogorov–Smirnov test. Numerical variables with a normal distribution were expressed as the mean and standard deviation. In the analysis of the categorical variables, the chi-squared test was used. The independent samples t test was used to compare numerical variables between the groups. The Pearson’s correlation coefficient was used to determine correlations between cerebrovascular aneurysmal haemorrhage and the CAVI parameters. p < 0.05 was considered statistically significant.

## 3. Results

The mean age of the 26 patients with cerebrovascular aneurysm was 55 ± 11 years, and 57.7% (n = 15) were female. In the cerebral aneurysmal haemorrhage group, the mean age was 58 ± 12 years, and 42.3% (n = 11) were female. No significant differences were found between the two groups in terms of the remaining demographic data, laboratory test results, systolic and diastolic blood pressure values, glomerular filtration, or mean ejection fraction (p > 0.05).

The mean aneurysm size was 8.3 ± 1.1 mm in the cerebrovascular aneurysm group and 8.7 ± 1.2 mm in the cerebrovascular aneurysmal haemorrhage group. There was no statistically significant difference between the groups in terms of the aneurysm size (p = 0.317). The laboratory results and demographic characteristics of the study patients are presented in Table 1.

The L-CAVI (7.89 ± 1.29 vs. 9.16 ± 2.07, p = 0.010, respectively) and R-CAVI (7.72 ± 1.35 vs. 9.45 ± 2.20, p = 0.020, respectively) measurements were significantly higher in the cerebrovascular aneurysmal haemorrhage group compared to the cerebrovascular aneurysm group (Table 2).

A positive correlation was observed between cerebral aneurysmal haemorrhage and arterial stiffness. This correlation was statistically significant in terms of the L-CAVI and R-CAVI. The R-CAVI showed a moderate correlation coefficient (r = 0.440, p = 0.002) (Table 3).

## 4. Discussion

Arterial stiffness was higher in patients with CAs who developed SAH compared to those who did not. This finding suggests that arterial stiffness measurements in patients with ruptured CAs may provide an idea about the risk of potential SAH and guide follow-up and treatment decisions.

The incidence of CAs is higher among women, individuals with a family history of SAH, and patients with autosomal dominant polycystic kidney disease [2]. Factors such as the patient’s age, aneurysm size, arterial hypertension, aneurysm localisation, and a previous history of SAH have been reported as independent risk factors for rupture [2]. In the current study, no significant differences were observed between the haemorrhage and nonhaemorrhage groups in terms of age, arterial hypertension, or sex. The lack of differences in these well-established risk factors underscores the importance of arterial stiffness as a potentially distinctive factor in assessing rupture risk.

Increased central and peripheral arterial stiffness has been previously shown in clinical studies as a marker associated with cardiovascular events and mortality [15,16]. Similarly, the CAVI assessment proposed herein may serve as a biomarker to evaluate the risk of IA rupture. Previous studies have linked increased arterial wall stiffness with a higher susceptibility to rupture in patients with CAs [17]. Stiffness is affected by the elastic properties of arterial wall tissue, and reduced elasticity has been associated with the development of SAH [17,18]. Arterial stiffness has also been linked to the degeneration of collagen, elastin, and the extracellular matrix [19]. Degeneration of vascular collagen contributes to arterial wall weakness by reducing wall elasticity, thereby increasing the likelihood of rupture [17,20]. Although the present study did not investigate the pathophysiological causes of arterial stiffness, the findings were consistent with previous studies correlating increased arterial stiffness with a heightened risk of SAH in patients with CAs.

The risk of rupture in abdominal aortic aneurysms has also been extensively investigated [21,22]. Studies have reported that vasculopathy and arterial distensibility predispose patients to the development, dissection, and rupture of abdominal aortic aneurysms [23]. In these patients, dissection occurs when wall tension exceeds the strength limit of the arterial wall [24]. Research evaluating the limit of aneurysm expansion has shown a correlation between the degree of distensibility and rupture risk [25,26]. Increased arterial wall stiffness, reduced elasticity, and decreased perivascular support may be associated with vasculopathies caused by ageing, essential hypertension, diabetes mellitus, and vasculitis [27,29]. Although the current study focused on CAs rather than abdominal aortic aneurysms, the consistent relationship between arterial stiffness and rupture risk underscores the general applicability of this phenomenon across different anatomical regions.

Cumulative arterial wall disruption due to continuous remodelling, characterised by degeneration and inflammatory cell infiltration, has been implicated in the pathogenesis of SAH [30]. The results herein suggest that increased stiffness of the arterial vasculature may lead to higher susceptibility to haemorrhage in patients with aneurysms.

The primary limitation of the study was the small sample size. In addition, the cross-sectional design precluded the establishment of causal relationships. The exclusion of posterior circulation aneurysms and aneurysms over 10 mm further limited the generalisability of the findings to all aneurysm subtypes. Therefore, large-scale studies that include diverse aneurysm types are needed to validate the results. Another limitation was the inability to predict changes in the CAVI values due to haemodynamic changes (e.g., nitric oxide levels) in patients with SAH. Despite these limitations, we consider that arterial stiffness is an important parameter in assessing CAs, which represent an isolated disease group with significant haemorrhage and mortality risks.

## 5. Conclusion

This study suggests that arterial stiffness evaluation in patients with CAs may provide valuable guidance in assessing the risk of SAH and may guide follow-up and treatment decisions.

## Figures and Tables

**Table 1:** Demographic and laboratory findings of the study group.

Variables	Cerebrovascular aneurysm (n = 26, 53.1%)	Cerebrovascular aneurysmal haemorrhage (n = 23, 46.9%)	p-value
**Age (years)**	55 ± 11	58 ± 12	0.308
**Sex, female (%)**	15 (57.7%)	11 (42.3%)	0.490
**HT (%)**	12 (52.2%)	11 (47.9%)	0.907
**BMI (kg/m** **^2^** **)**	28.3 ± 6.5	27.9 ± 4.9	0.751
**SBP (mmHg)**	137 ± 8	134 ± 7	0.172
**DBP (mmHg)**	83 ± 3	80 ± 2	0.138
**EF (%)**	59 ± 3	58 ± 2	0.358
**Cerebral aneurysm size (mm)**	8.3 ± 1.1	8.7 ± 1.2	0.317
**Creatinine, mg/dl**	0.9 ± 0.1	0.9 ± 0.3	0.543
**eGFR, mL/min/1.73 m2**	75 ± 5	72 ± 3	0.687
**Glucose (mg/dl)**	98 ± 18	93 ± 12	0.215
**Haemoglobin, g/dl**	13.1 ± 1.3	12.8 ± 2.1	0.546
**Total cholesterol (mg/dl)**	191 ± 56	185 ± 42	0.652
**LDL (mg/dl)**	120 ± 39	114 ± 30	0.571

Numerical data are expressed as the mean ± standard deviation. BMI: body mass index, SBP: systolic blood pressure, DBP: diastolic blood pressure, EF: ejection fraction, eGFR: estimated glomerular filtration, LDL: low-density lipoprotein.

**Table 2:** Comparison of the CAVI findings between the study groups.

Variables	Cerebrovascular aneurysm (n = 26, 53.1%)	Cerebrovascular aneurysmal haemorrhage (n = 23, 46.9%)	p value
**L-CAVI**	7.85 ± 1.29	9.16 ± 2.07	0.010
**L-ABI**	1.04 ± 0.06	0.99 ± 0.14	0.143
**R-CAVI**	7.72 ± 1.35	9.45 ± 2.20	0.002
**R-ABI**	1.03 ± 0.07	1.01± 0.09	0.514

L: left, R: right, CAVI: cardio-ankle vascular index, ABI: ankle-brachial index.

**Table 3:** Correlation analysis results of the cerebrovascular aneurysmal haemorrhage and CAVI.

Variables	Cerebrovascular aneurysmal haemorrhage	
	R	p*-*value
**L-CAVI**	0.366	0.010
**R-CAVI**	0.440	0.002

## References

[1] Brown RD Broderick JP Unruptured intracranial aneurysms: epidemiology, natural history, management options, and familial screening The Lancet Neurology 2014 13 4 393 404 10.1016/S1474-4422(14)70015-8 24646873

[2] Hackenberg KA Hänggi D Etminan N Unruptured intracranial aneurysms: contemporary data and management Stroke 2018 49 9 2268 2275 10.1161/STROKEAHA.118.02103 30355003

[3] Frösen J Tulamo R Paetau A Laaksamo E Korja M Saccular intracranial aneurysm: pathology and mechanisms ActaNeuropathologica 2012 123 773 786 10.1007/s00401-011-0939-3 22249619

[4] Thompson BG Brown JRD Amin-Hanjani S Broderick JP Cockroft KM Guidelines for the management of patients with unruptured intracranial aneurysms: a guideline for healthcare professionals from the American Heart Association/American Stroke Association Stroke 2015 46 8 2368 2400 10.1161/STR.0000000000000070 26089327

[5] Jayaraman T Paget A Shin YS Li X Mayer J TNF-alpha-mediated inflammation in cerebral aneurysms: a potential link to growth and rupture Vascular Health and Risk Management 2008 4 805 817 10.2147/vhrm.s2700 19065997 PMC2597764

[6] Tulamo R Frösen J Junnikkala S Paetau A Pitkäniemi J Complement activation associates with saccular cerebral artery aneurysm wall degeneration and rupture Neurosurgery 2006 59 1069 1076 10.1227/01.NEU.0000245598.84698.26 17016232

[7] Sakata N Takebayashi S Shimizu K Kojima M Masawa N A case of segmental mediolyticarteriopathy involving both intracranial and intraabdominal arteries Pathology - Research and Practice 2002 198 493 497 10.1078/0344-0338-00290 12234069

[8] Giannarelli C Bianchini E Bruno RM Magagna A Landini L Local carotid stiffness and intimamedia thickness assessment by a novel ultrasound-based system in essential hypertension Atherosclerosis 2012 223 372 377 10.1016/j.atherosclerosis.2012.05.027 22727194

[9] Rogowicz FA Araszkiewicz A Pilacinski S Zozulinska ZD Wykretowicz A Carotid intima-media thickness and arterial stiffness in type 1 diabetic patients with and without microangiopathy Archives of Medical Science 2012 8 484 490 10.1055/s-0030-1267184 22852004 PMC3400914

[10] Akashi R Koga S Yonekura T Ikeda S Kawano H Cardio-ankle vascular index is associated with coronary plaque composition assessed with iMAP-intravascular ultrasound in patients with coronary artery disease Journal of Cardiology 2021 78 6 502 508 10.1016/j.jjcc.2021.05.015 34284942

[11] Shirai K Hiruta N Song M Kurosu T Suzuki J Cardio-ankle vascular index (CAVI) as a novel indicator of arterial stiffness: theory, evidence and perspectives Journal of Atherosclerosis and Thrombosis 2011 18 11 924 938 21628839 10.5551/jat.7716

[12] Shirai K Utino J Otsuka K Takata M A novel blood pressure-independent arterial wall stiffness parameter; cardio-ankle vascular index (CAVI) Journal of Atherosclerosis and Thrombosis 2006 13 2 101 107 10.5551/jat.13.101 16733298

[13] Takahashi K Yamamoto T Tsuda S Okabe F Shimose T Coefficients in the CAVI equation and the comparison between CAVI with and without the coefficients using clinical data Journal of atherosclerosis and thrombosis 2019 26 5 465 475 10.5551/jat.44834 30518727 PMC6514175

[14] Stoffers HE Kester AD Kaiser V Rinkens PE Kitslaar PJ The diagnostic value of the measurement of the ankle–brachial systolic pressure index in primary health care Journal of Clinical Epidemiology 1996 49 1401 1405 10.1016/S0895-4356(96)00275-2 8970490

[15] Vlachopoulos C Aznaouridis K Stefanadis C Prediction of cardiovascular events and all-cause mortality with arterial stiffness: a systematic review and meta-analysis Journal of the American College of Cardiology 2010 30 55 13 1318 1327 10.1016/j.jacc.2009.10.061 20338492

[16] Ben SY Spears M Boustred C May M Anderson S Aortic pulse wave velocity improves cardiovascular event prediction: an individual participant meta-analysis of prospective observational data from 17,635 subjects Journal of the American College of Cardiology 2014 63 636 646 10.1016/j.jacc.2013.09.063 24239664 PMC4401072

[17] Costalat V Sanchez M Ambard D Thines L Lonjon N Biomechanical wall properties of human intracranial aneurysms resected following surgical clipping Journal of Biomechanics 2011 44 2685 2691 10.1016/j.jbiomech.2011.07.026 21924427

[18] Maltete D Bellien J Cabrejo L May M Iacob M Hypertrophic remodeling and increased arterial stiffness in patients with intracranial aneurysms Atherosclerosis 2010 211 486 491 https:///10.1016/j.atherosclerosis.2010.04.002 20452592 10.1016/j.atherosclerosis.2010.04.002

[19] Wilson KA Lindholt JS Hoskins PR Heickendorff L Vammen S The relationship between abdominal aortic aneurysm distensibility and serum markers of elastin and collagen metabolism European Journal of Vascular and Endovascular Surgery 2001 21 175 178 10.1053/ejvs.2001.1303 11237793

[20] Wilson KA Lee AJ Lee AJ Hoskins PR Fowkes FGR The relationship between aortic wall distensibility and rupture of infrarenal abdominal aortic aneurysm Journal of Vascular Surgery 2003 37 112 117 10.1067/mva.2003.40 12514586

[21] Van’t Veer M Buth J Merkx M Tonino P Van den Bosch H Biomechanical properties of abdominal aortic aneurysms assessed by simultaneously measured pressure and volume changes in humans Journal of Vascular Surgery 2009 48 6 1401 1407 10.1016/j.jvs.2008.06.060 18771885

[22] Evrengul H Tanriverdi H Kilic ID Dursunoglu D Ozcan EE Aortic stiffness and flow-mediated dilatation in normotensive offspring of parents with hypertension Cardiology in The Young 2012 22 4 451 456 10.1017/S104795111200008X 22348859

[23] Van Laake W Vainas T Dammers R Kitslaar PJEHM Hoeks APG Systemic dilation diathesis in patients with abdominal aortic aneurysms: a role for matrix metalloproteinase-9? European Journal of Vascular and Endovascular Surgery 2005 29 4 371 377 10.1016/j.ejvs.2005.01.009 15749037

[24] Wilson KA Lee AJ Hoskins PR Fowkes FGR Ruckley CV The relationship between aortic wall distensibility and rupture of infrarenal abdominal aortic aneurysm Journal of Vascular Surgery 2003 37 1 112 117 10.1067/mva.2003.40 12514586

[25] Wilson KA Lindholt JS Hoskins PR Heickendorff L Vammen S The relationship between abdominal aortic aneurysm distensibility and serum markers of elastin and collagen metabolism European Journal of Vascular and Endovascular Surgery 2001 21 2 175 178 10.1053/ejvs.2001.1303 11237793

[26] Gaal EI Salo P Kristiansson K Rehnström K Kettunen J Intracranial aneurysm risk locus 5q23. 2 is associated with elevated systolic blood pressure PLoS Genetics 2012 8 3 e1002563 10.1371/journal.pgen.1002563 22438818 PMC3305343

[27] Greene ER Lanphere KR Sharrar J Roldan CA Arterial distensibility in systemic lupus erythematosus Medicine and Biology Society 2009 1109 1112 10.1109/IEMBS.2009.5334459 PMC3631272 19964750

[28] Zapolski T Wysokiński A Left atrium volume index is influenced by aortic stiffness and central pulse pressure in type 2 diabetes mellitus patients: a hemodynamic and echocardiographic study Medical Science Monitor 2013 19 153 164 10.12659/MSM.883818 23458774 PMC3628717

[29] Orlova IA Nuraliev EY Yarovaya EB Ageev FT Prognostic value of changes in arterial stiffness in men with coronary artery disease Vascular Health and Risk Management 2010 6 1015 1021 10.2147/VHRM.S13591 21127698 PMC2988619

[30] Tulamo R Frösen J Junnikkala S Paetau A Pitkäniemi J Complement activation associates with saccular cerebral artery aneurysm wall degeneration and rupture Neurosurgery 2006 59 1069 1076 10.1227/01.NEU.0000245598.84698.26 17016232

